# Changes in alcohol consumption in the Russian Federation during the first months of the COVID-19 pandemic

**DOI:** 10.1192/j.eurpsy.2022.1258

**Published:** 2022-09-01

**Authors:** A. Gil, K. Vyshinsky, E. Fadeeva, R. Khalfin

**Affiliations:** 1 I.M. Sechenov First Moscow State Medical University (Sechenov University), Higher School Of Health Administration, Moscow, Russian Federation; 2 National Research Centre on Addictions – branch, Federal State Budgetary Institution «National Medical Research Center for Psychiatry and Narcology named after VP Serbsky» of the Ministry of Health of the Russian Federation, Epidemiology Department, Moscow, Russian Federation; 3 National Medical Research Centre for Psychiatry and Narcology n.a. V.Serbsky Russian Federation Ministry of Health, Head Of Department Of Preventive Care In Narcology, Moscow, Russian Federation

**Keywords:** Russia, alcohol, Covid-19, alcohol consumption

## Abstract

**Introduction:**

Excessive alcohol consumption is a known risk factor for various mental health disorders and can exacerbate the already high burden of COVID-19 pandemic on mental health.On the other hand, the COVID-19 pandemic itself can adversely affect alcohol consumption and thus contribute to alcohol-related problems, including mental health problems.

**Objectives:**

This study was aimed to assess changes in alcohol consumption that may have occurred as a result of the COVID-19pandemic and determine associated factors among population of Russian Federation.

**Methods:**

By distributing a link to take part in an anonymous online survey,changes in volume and frequency of alcohol use,and frequency of heavy episodic drinking(6 or more servings of alcohol at a time)in the first months of COVID-19pandemic were assessed. 819respondents from Russia:321 men and 498women, submitted their responses during May-July,2020. Associations between changes in alcohol use were assessed in a univariate analysis with socio-demographic factors,alcohol use over the previous 12months,stress, individual perceptions of changes in daily and social life and other negative consequences of pandemic.The statistical significance of associations was assessed using the Pearson’sχ2 test.

**Results:**

Individuals with initially higher alcohol consumption increased their alcohol use, while those who drank less, decreased alcohol use even more during pandemic (p<0.05). Severe restrictions of social/everyday life were associated with more frequent alcohol use and in larger volumes (p<0.001). Negative professional/financial consequences of pandemic and stress were associated with increase of typical drinking volume (p<0.001), more frequent alcohol use (p<0.001)and heavy episodic drinking (p<0.05).

**Conclusions:**

The COVID-19 pandemic could have increased health inequalities in Russia through changes in alcohol consumption.

**Disclosure:**

No significant relationships.

